# Multifunctional Protein A Is the Only Viral Protein Required for Nodavirus RNA Replication Crown Formation

**DOI:** 10.3390/v14122711

**Published:** 2022-12-03

**Authors:** Johan A. den Boon, Hong Zhan, Nuruddin Unchwaniwala, Mark Horswill, Kailey Slavik, Janice Pennington, Amanda Navine, Paul Ahlquist

**Affiliations:** 1 John W. and Jeanne M. Rowe Center for Research in Virology, Morgridge Institute for Research, Madison, WI 53715, USA; 2 Institute for Molecular Virology, University of Wisconsin–Madison, Madison, WI 53706, USA; 3 McArdle Laboratory for Cancer Research, University of Wisconsin–Madison, Madison, WI 53705, USA

**Keywords:** positive-strand RNA virus, nodavirus, RNA replication complex, cryo-EM tomography, crown complex

## Abstract

Positive-strand RNA virus RNA genome replication occurs in membrane-associated RNA replication complexes (RCs). Nodavirus RCs are outer mitochondrial membrane invaginations whose necked openings to the cytosol are “crowned” by a 12-fold symmetrical proteinaceous ring that functions as the main engine of RNA replication. Similar protein crowns recently visualized at the openings of alphavirus and coronavirus RCs highlight their broad conservation and functional importance. Using cryo-EM tomography, we earlier showed that the major nodavirus crown constituent is viral protein A, whose polymerase, RNA capping, membrane interaction and multimerization domains drive RC formation and function. Other viral proteins are strong candidates for unassigned EM density in the crown. RNA-binding RNAi inhibitor protein B2 co-immunoprecipitates with protein A and could form crown subdomains that protect nascent viral RNA and dsRNA templates. Capsid protein may interact with the crown since nodavirus virion assembly has spatial and other links to RNA replication. Using cryoelectron tomography and complementary approaches, we show that, even when formed in mammalian cells, nodavirus RC crowns generated without B2 and capsid proteins are functional and structurally indistinguishable from mature crowns in infected *Drosophila* cells expressing all viral proteins. Thus, the only nodaviral factors essential to form functional RCs and crowns are RNA replication protein A and an RNA template. We also resolve apparent conflicts in prior results on B2 localization in infected cells, revealing at least two distinguishable pools of B2. The results have significant implications for crown structure, assembly, function and control as an antiviral target.

## 1. Introduction

The positive-strand (+)RNA viruses include many important human, animal, and plant pathogens. Their genomes are mRNA-sense single-stranded RNAs whose replication process occurs in the host cell cytoplasm with no intermediate DNA stage. Without exception, such (+)RNA viruses rearrange host intracellular membranes to create new compartments that serve as their RNA genome replication complexes (RC), concentrating viral and host factors, organizing the various successive steps in the RNA replication process, and preventing recognition by host antiviral defense mechanisms [1,2,3]. The most common type of these compartments are necked vesicles or spherules that are invaginated into a target membrane whose nature varies with different viruses from organellar and secretory to plasma membranes. 

Such spherule RCs have been analyzed particularly well for a small number of (+)RNA viruses including nodaviruses, a growing family of (+)RNA viruses of invertebrates and vertebrates. While virions of the well-studied nodavirus Flock House nodavirus (FHV) are naturally only infectious to insect cells, when introduced by other means, the FHV genome will launch RNA replication, transcription and virion assembly in many eukaryotic cells, including mammalian, plant, fungal and nematode cells [4,5,6]. Because of this and other advantageous features, FHV has become an advanced model for (+)RNA virus genome replication [7,8].

**Figure 1:**
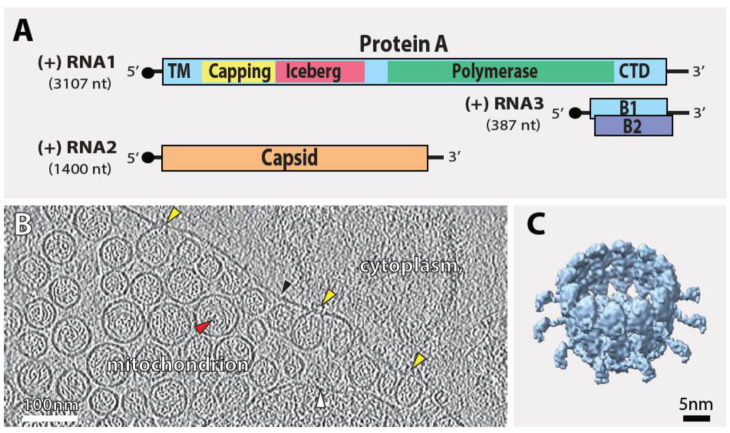
Nodavirus genome organization and replication. (**A**) The bipartite FHV RNA genome encodes the viral replicase protein A, the key protein for viral RNA replication, on RNA1 and the capsid protein precursor on RNA2. Subgenomic RNA3, transcribed from RNA1, encodes protein B1, identical to the C-terminus of protein A, and protein B2 which is an RNAi suppressor. (**B**) Cryo-EM tomogram of Drosophila S2 cells with nodavirus replication complexes: invaginations (spherules) of the outer mitochondrial membrane into a vastly dilated intermembrane space. Arrowheads: black = outer mitochondrial membrane; white = inner mitochondrial membrane; red = spherules, filled with dsRNA; yellow = crown-like structures at the spherule open necked connections to the cytoplasm. (**C**) Tomographic reconstruction of the 12-fold symmetrical nodavirus replication complex crown, from reference [24].

Nodaviruses have a bipartite genome (Figure 1A). Genomic RNA1 encodes the highly multifunctional RNA replicase, the 112 kDa protein A. Protein A has an N-terminal mitochondrial membrane association domain, RNA methyltransferase (MTase)/capping domain, RNA-dependent RNA polymerase (RdRp) and a C-terminal, ~100 aa extension predicted to be structurally disordered with no currently defined function. Throughout the protein, there are multiple self-interaction domains that drive its multimerization [9]. Genomic RNA1 is also the template for producing a subgenomic RNA, RNA3, which encodes two small, ~14 kDa proteins: protein B1, co-linear with protein A’s C-terminus, is a nuclear protein that at least in cell culture is dispensable for FHV replication [10,11]. In a separate reading frame, RNA3 encodes protein B2, a dsRNA-binding protein that strongly inhibits host RNA interference [12,13]. RNA2 encodes the capsid protein that in the later stages of the viral life cycle packages the progeny genomic RNAs 1 and 2 into a single virion.

Nodaviruses target their RCs to the outer mitochondrial membrane (OMM) using the N-proximal mitochondrial transmembrane domain in protein A [14]. Further membrane interactions are predicted to be mediated through sequences in a region in protein A downstream from MTase region resembling the “Iceberg” region in alphavirus replicases (Figure 1A) [15]. Protein A recruits genomic RNA1 to mitochondria through interactions between its RdRp domain and cis-acting RNA1 signals (nt 68–205) [16,17]. Combined with several lateral interactions between individual copies of protein A [9], these and further events lead to the induction of characteristic ~60–70 nm spherular invaginations of the OMM (Figure 1B) [14,18,19,20,21]. These “spherule” membrane invaginations provide the framework of the nodavirus RC, which initially replicates RNA1 and transcribes subgenomic RNA3. RNA3 accumulation subsequently transactivates replication of the second genomic RNA, RNA2, providing a mechanism to delay capsid production and virion assembly until after RNA replication is well-established [22]. 

Classical electron microscope (EM) tomography [19] and more recently cryo-electron tomography (cryo-ET) [23,24] of FHV-infected *Drosophila* cells provided the first three-dimensional views of a (+)RNA virus RC in increasing scope and resolution. The results show that nodavirus RC spherular membrane invaginations are continuous with the outer mitochondrial membrane [19,25]. These spherules are densely packed with filaments, consistent with extensive biochemical evidence that they contain the dsRNA replication intermediates, and spherule volume calculations imply that most spherules contain one or at most two dsRNAs [23]. Each spherule retains an open necked connection to the cytosol [19,25], gated by a 12-fold symmetric crown-like protein complex containing viral protein A [23]. These crown structures have more recently been resolved to ~8.5 Å, showing a central turret of twelve stacked apical and basal lobes surrounded by twelve outward leg-like extensions (Figure 1C) [24]. Each of the twelve units in the crown has two apparent interactions with the outer mitochondrial membrane: one at the base of the central turret and another at each leg, which together appear critical to maintain the strong curvature of the RC vesicle neck. These membrane interactions must include protein A’s N-terminal membrane-spanning mitochondrial anchor and, by immunogold labeling, the 12 apical lobes of the crown’s central turret have been identified as copies of protein A’s RdRP domain [24].

While it is thus clear that protein A is a major, high copy number constituent of the crown, all crown structures resolved to date have been derived from FHV-infected cells and the nature of most crown domains remains unknown. Accordingly, besides multiple copies of protein A, the crown might include additional viral proteins, with prime candidates being the capsid and B2 proteins to account for prior results linking encapsidation and RNAi suppression to RNA replication (see below). Here, using genetically modified derivatives of genomic RNA1, cryo-ET and complementary approaches, we show that the capsid and B2 proteins are dispensable for generating wildtype crown structures, indicating that protein A is the sole viral protein component of the crown.

## 2. Materials and Methods

### 2.1. Cell Culture, Virus Infection

*Drosophila* S2 cells were maintained at 28 °C in Express Five medium supplemented with 120 U/mL penicillin, 100 µg/mL streptomycin and 2 mM L-glutamine (PSG). BSRT7/5 cells were maintained at 37 °C under 5% CO_2_ and maximum humidity in DMEM supplemented with 8% bovine calf serum (BCS) and PSG. FHV infections used an m.o.i. of 5 at room temperature for 30–60 min, were further incubated at 28 °C, and typically harvested at 16 to 18 h post-infection. 

### 2.2. Plasmid Construction

Standard molecular cloning procedures were used to place FHV RNA1 and derivative sequences under control of the baculovirus IE1 promoter for expression in *Drosophila* S2 cells or the bacteriophage T7 promoter for expression in mammalian BSRT7/5 cells, both with a downstream hepatitis delta virus ribozyme sequence (plasmids pIE1hr5-FHVRNA1Rz, pIE1hr5-FHVRNA1Rz-noB2, pT7-FHVRNA1Rz, and pT7-FHVRNA1Rz-noB2). Oligonucleotides and synthetic DNA gBLOCKs were purchased from IDT DNA Technologies, Coralville, IA, USA. Plasmid sequences were verified using BigDye Sanger sequencing and analysis at the UW-Madison Biotechnology Center Core Sequencing Facilities.

### 2.3. In Vitro Transcription & Capping

For in vitro transcription, plasmids were linearized at a suitable run-off position, proteinase K-treated, phenol/chloroform/isoamyl-alcohol-extracted, and ethanol-precipitated. Linearized DNA templates were used for T7-polymerase-mediated transcription using AmpliScribe T7-Flash Transcription kit (Lucigen, Madison, WI, USA). ScriptCap m7G Capping System (CELLSCRIPT, Madison, WI, USA) was used to add 5’ m^7^G caps to the RNA transcripts. Both before and after capping, New England Biolabs (Ipswich, MA, USA), Monarch Total RNA miniprep kit was used to clean up RNA transcripts.

### 2.4. DNA and RNA Transfection

For transfection of 3 × 10^6^ Drosophila S2 cells in six-well plate tissue culture format, 1 µg DNA plasmid was complexed with 4 µL Lipofectamine^TM^3000 (ThermoFisher Scientific, Waltham, MA, USA) and 4 µL P3000 transfection reagent in 100 µL Opti-MEM, incubated for 15 min at room temperature prior to addition to 5 × 10^6^ cells. S2 transfections were incubated at 28 °C and harvested at 40–44 h post-transfection. RNA transfections in BSRT7/5 cells were mediated using 4 µL Trans-IT Insect transfection reagent (MIRUS-Bio, Madison, WI, USA) per 1 µg in vitro transcribed/capped RNA per six-well plate well containing 3 × 10^6^ cells. BSRT7/5 transfections were incubated for 3 h at 37 °C, transferred to 32 °C, and harvested at 40–44 h post-transfection. Transfections were scaled as needed according to tissue culture vessel size.

### 2.5. Western Blotting

Total protein was extracted from 1 × 10^6^ cells using 200 µL preheated (85 °C) Cracking Buffer (8M Urea, 5% SDS, 40 mM TrisHCl pH 7.0, 100 mM EDTA, 25% glycerol, and a trace of orange G). Samples were heated to 85 °C for 10 min and 10–15 µL were resolved in NuPAGE Novex 4–12% or 8% Bis-Tris Midi Protein denaturing SDS PAGE gels (ThermoFisher Scientific) using 1× NuPAGE MOPS SDS running buffer. Upon completion, at room temperature, gels were washed in ddH_2_O for 10 min, equilibrated in 2× NuPAGE transfer buffer for 15 min and proteins were transferred to methanol-soaked Immobilon-FL PVDF membrane equilibrated in 2× NuPAGE transfer buffer using semi-dry blotting for 10 min at 25 V. Blots were blocked in Odyssey TBS Blocking Buffer (LI-COR, Lincoln, NE, USA) for 1 h and incubated with primary antibody, typically at 1:1000 to 1:5000 dilution, in TBS supplemented with 0.2% Tween20 for 1–4 h. At room temperature, blots were washed three times in TBS with 0.1% Tween20 for 5 min, incubated for 1 h with secondary antibody IRDye 680RD or 800CW (1:10,000) in TBS with 0.2% Tween20 and 0.01% SDS, washed three times in TBS with 0.1% Tween20 for 5 min, and rinsed with TBS prior to imaging on a LI-COR Odyssey imaging system.

### 2.6. Northern Blotting

Total RNA was extracted using Trizol (Invitrogen, Waltham, MA, USA) at 1 mL per 1–2.5 × 10^6^ cells. RNA samples (typically 500 ng) were mixed 1:2 (*v*/*v*) with RNA sample loading buffer (Sigma, St. Louis, MO, USA; R4268-5VL), and resolved in a 1.5% denaturing agarose gel containing running buffer (20 mM MOPS, 8 mM sodium acetate, 1 mM EDTA), supplemented with 2% formaldehyde. RNA was transferred to Biodyne (Fort Wayne, IN, USA) B Nylon membranes using overnight passive upward transfer mediated by 10 × SSPE (1.5M NaCl, 0.1M NaH_2_PO_4_, 0.01M EDTA, pH 7.4). Upon transfer, blots were rinsed in 5 × SPPE and crosslinked twice using auto-settings in a UVP CL-1000 UV crosslinker. RNA blots were pre-hybridized in NorthernMAX solution (Ambion, Austin, TX, USA) for 1 h at 42 °C, and refreshed with NorthernMAX solution containing 250–500 ng biotinylated RNA probe for a minimum of 3 h at 42 °C. RNA probes were generated by in vitro T7 transcription of 1 µg linearized plasmid template using a MAXIscript kit (Ambion) for 1 h at 37 °C using nucleotide concentrations at 0.5 mM for ATP, CTP, and GTP, 0.3 mM for UTP, and 0.2 mM for Bio-16-UTP, followed by DNaseI-treatment for 15 min at 37 °C and ethanol-precipitation. Upon hybridization, blots were rinsed in high-salt buffer (2× SSPE, 0.2% SDS), washed for 20 min in low-salt buffer (0.2× SSPE, 0.2% SDS) at 42 °C, blocked using Odyssey PBS blocking buffer (LI-COR) containing 1% SDS for 1 h at room temperature, and incubated in a 1:10,000 dilution of either IRDye680RD-streptavidin or IRDye800CW-streptavidin in blocking buffer containing 1% SDS for 30 min at room temperature, washed three times in PBS with 0.1% Tween20, and once in PBS prior to imaging using a LI-COR Odyssey system.

### 2.7. Immunofluorescence

BSRT7/5 cells grown on glass coverslips (Neuvitro, Vancouver, WA, USA; GG-18-PDL) were washed three times with PBS w/o Ca^2+^ and Mg^2+^ (Corning, NY, USA; 21-040-CM, for all subsequent washes), fixed in 4% paraformaldehyde in PBS for 10 min at room temperature, washed three times in PBS, permeabilized with 0.2% Triton X-100 in PBS for 10 min, and washed three times for 5 min in PBS. At room temperature, cells were incubated in blocking solution (50 mM NH_4_Cl, 2% BSA, 0.05% N_3_Na in PBS) for 1 h, incubated with primary antibodies in blocking solution for 1 h, washed three times in PBS, incubated for 1 h with secondary antibodies in blocking solution, and washed three times for 5 min in PBS. Primary antibodies were polyclonal rabbit-anti-FHV protein A (R1194) at 1:500, mouse monoclonal anti-FHV protein B2 (monoclonal clone 2A-8) at 1:500, and mouse monoclonal anti-dsRNA (Scicons, Nordic MUbio, Susteren, The Netherlands; clone J2). Secondary antibodies were goat-anti-rabbit AlexaFluor 568 (ThermoFisher, A11011), goat-anti-mouse AlexaFluor 647 (ThermoFisher A21235), and donkey-anti-mouse AlexaFluor 647 (ThermoFisher, A31571), at 1:200 dilution. Coverslips were incubated with NucBlue (Invitrogen, 2 drops diluted in 1 mL PBS)) for 5 min at room temperature, washed three times for 2 min in PBS, drained, and mounted using 20 µL ProLong Glass Antifade Mountant (Invitrogen) and cured for at least 1–2 h in the dark at room temperature. Slides were imaged using either a Zeiss ELYRA SIM microscope at the Newcomb Imaging Center or a wide-field epifluorescence Nikon Ti microscope. Image processing used the Fiji (ImageJ) software package.

### 2.8. Immunoprecipitation

To detect interaction between FHV proteins A and B2, approximately 6 × 10^7^ FHV- or mock-infected S2 cells were harvested 16 h post-infection and lysed in 1300 µL ice-cold TBS-N (50 mM Tris-HCl pH7.5, 150 mM NaCl), 1 mM EDTA, 2 mM ß-mercaptoethanol, 0.5% NP-40) containing protease inhibitors for 10 min on ice. Nuclei were pelleted at 10,000× *g* for 5 min at 4 °C. A small supernatant sample was set aside for total protein analysis, and 250 µL aliquots were adjusted to 500 µL with TBS-N and incubated with either 10 µL rabbit anti-FHV protein A antibody (R1194) or no antibody for 1 h at 4 °C. 25 µL protein A/G magnetic beads (PIERCE) were added and mixtures were incubated o/n at 4 °C. Beads were washed three times in TBS-N and samples were adjusted to 5 mM CaCl_2_. Either 1 µL micrococcal nuclease was added or no nuclease as a control. Samples were incubated at 37 °C for 30 min, washed three times in TBS-N, and protein was released from the beads in 250 µL Cracking Buffer at 90 °C for 10 min prior to SDS PAGE and Western blotting.

### 2.9. Preparation of Mitochondria for Cryo-Electron Microscopy Imaging

High-purity sub-cellular mitochondrial fractions were obtained from 4–5 × 10^7^ cells using a Qproteome Mitochondria Isolation Kit (Qiagen, Hilden, Germany). A few µl of such mitochondrial preparations were deposited on 200 mesh Quantifoil cryogrids (R2/2) (Electron Microscopy Sciences, Hatfield, PA, USA; Q2100CR2,) that had been glow-discharged for 60 s using PELCO easiGlow (Ted Pella, Inc., Redding, CA, USA). Grids were plunge-frozen using a Vitrobot (FEI, ThermoFisher) under 100% humidity and 22 °C using fresh blotting paper (Ted Pella, Inc.) for each sample. Freezing parameters have been previously described [24]. One grid from each set of frozen samples was pre-screened on a Tecnai F30 (ThermoFisher, Inc.) equipped with a Gatan K2 summit direct electron detector and a post-column energy filter (Gatan, Inc., Pleasanton, CA, USA) and operated at an accelerated voltage of 300 KeV and with sliter width of 20 eV. Samples with sufficient quality based on mitochondrial density and ice thickness were shipped to the HHMI Janelia Farms Research Campus CryoEM center for imaging and tilt-series acquisition.

### 2.10. Tomogram Tilt-Series Acquisition and Sub-Tomogram Averaging

Samples were imaged using a Titan Krios operated at 300 KeV equipped with a K2 Summit direct electron detector and a post-column energy filter (Gatan, Inc) with sliter width of 20 eV at the Janelia Research Campus CryoEM center. Tilt series were collected using UCSFtomo [26] at a tilt range from −60° to 60° with 2° increments. The accumulated total dose for a tilt-series was at 180 e-/Å^2^ and a bidirectional tilt acquisition scheme was performed at 19,500× magnification, resulting in a pixel size of 5.4 Å. Each tilt was fractionated to 12 frames in 3.7 s exposure time at accumulated dose rate 2.95 e-/Å^2^. Individual tilt-containing frames were corrected for drift using Unblur from *Cis*TEM [23]. Tilt-series for subsequent sub-tomogram averaging were selected based on reconstruction quality and automatically transferred to the UW-Madison Center for High-Throughput computing (CHTC). Using PEET v1.15/IMOD [27,28], crowns were selected using an open contour with 2 points, and initial rotation parameters were generated automatically using stalkInit function. We manually picked 223 crown-like densities from one tomogram of mitochondria from wildtype RNA1-transfected BSRT7/5 cells and 2417 crown-like densities from 32 tomograms of the mitochondria from RNA1-noB2-transfected BSRT7/5 cells. Initial reconstructions with no symmetry imposed showed clear 12-fold symmetry and resolution was further improved by enforcing C12 symmetry in the final structure. UCSF ChimeraX [29] was used for all structure visualization, including low-pass Gaussian volume filtering to allow comparisons to our earlier published crown structure from nodavirus infected cells [24].

## 3. Results

As noted in the Introduction, FHV protein A localizes to OMMs where it recruits viral RNA templates, induces spherule RCs and provides essential enzymatic functions for RNA synthesis [14,16,17,20]. In keeping with these functions, immunogold labeling shows that protein A is present in the protein crowns of FHV RC spherules [23] as a repeated, 12-fold symmetric component [24]. Given protein A’s large size (998 aa) and high copy number, volume calculations show that protein A must comprise a large fraction of the crown [24]. Nevertheless, all nodavirus RC crown structures to date have been generated from FHV infections expressing the full complement of viral factors [23,24], so that the contribution of other viral factors to the crown structure has not been excluded.

Of the remaining nodaviral proteins, B1 is not only dispensable for RNA replication but localizes to the nucleus and not at the cytoplasmic sites of FHV RNA replication [10,11]. Moreover, RNA1 alone can induce RNA replication, showing that RNA2 and its encoded capsid protein are not required for RNA1 replication and subgenomic RNA3 transcription [20]. RNAi inhibitor protein B2 likewise is dispensable if RNAi is suppressed by other means [20]. However, RC crown structures have not been studied under these conditions to date and the B2 and capsid proteins remain prime candidates for participating in wildtype crowns, since nodavirus virion assembly has been linked to RNA replication and virions localize closely with viral RNA replication compartments [30,31], and B2 has been reported to interact with protein A in infected cells [32]. In the following sections we integrate multiple approaches culminating in cryo-EM to resolve these and related questions concerning crown structure and function. 

### 3.1. Small Subsets of Protein A and Protein B2 Interact in Infected Cells

**Figure 2:**
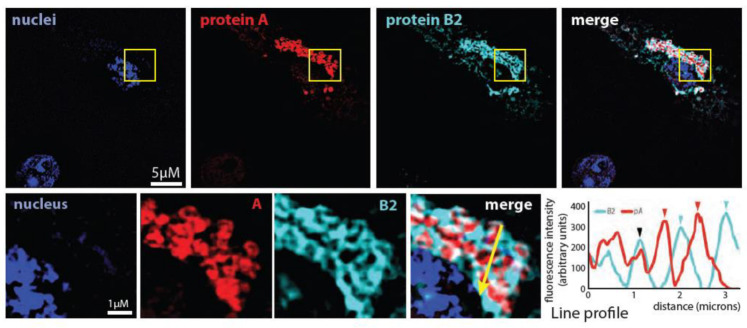
Subcellular localization of nodavirus proteins A and B2 in infected cells. Super-resolution Structural Illumination Microscopy (SIM) reveals that in nodavirus-infected cells, protein B2 localizes near the sites of protein A at mitochondria. Higher magnification insets show that protein A and B2’s localization is predominantly at distinct adjacent sites and that even at the resolution of light microscopy only small portions of their distributions overlap, as further demonstrated by fluorescence intensity measurements across a selected line profile (yellow arrow) in the lower right panels. Blue and red arrowheads indicate separate, distinct localization of proteins B2 and A, respectively; a black arrowhead indicates their occasional co-localization.

In prior support for a possible structural role for protein B2 in crown formation, Aliyari and co-workers reported that B2 co-immunoprecipitates with protein A [32]. However, Jovel and Schneemann used immunofluorescence to show that B2 accumulates at positions adjacent to, but not directly colocalized with, protein A at the mitochondria in FHV-infected cells [33]. In hope of reconciling these seemingly contradictory findings, we decided to independently verify these earlier reports. Accordingly, we first conducted structured illumination microscopy (SIM) immunofluorescence (IF) imaging using antibodies against protein A and protein B2. As shown in Figure 2, the B2 signal in FHV-infected *Drosophila* S2 cells was easily detectable and occupied spaces interspersed between and surrounding protein A at its well-established mitochondrial localization sites. Confirming Jovel and Schneemann’s observations, visual inspection of the merged super-resolution SIM images of protein A and protein B2 (lower magnified images) and fluorescence intensity line profile measurements in those images (lower right panel) showed that the peaks of B2 and protein A intensity did not coincide but rather directly alternated with each other across the image. 

**Figure 3:**
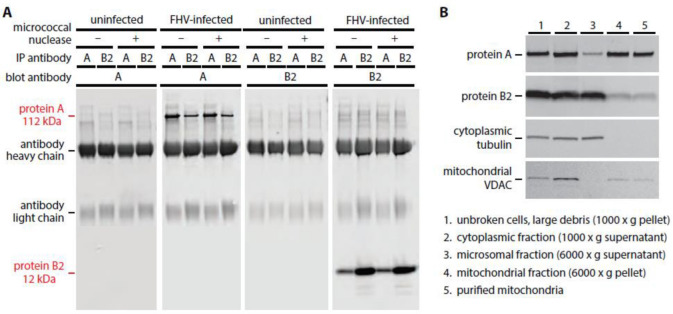
Small amounts of protein B2 interact with protein A and segregate with purified mitochondria. (**A**) Subsets of nodavirus proteins A and B2 physically interact. Antibodies directed against protein A co-immunoprecipitate protein B2 and antibodies directed against protein B2 co-immunoprecipitate protein A. The interaction is insensitive to micrococcal nuclease treatment, showing that it is not mediated through RNA. (**B**) Cell fractionation procedures to isolate mitochondria show that the majority of protein A but only a small amount of protein B2 (~8%) tracks with mitochondrial marker VDAC, while the great bulk of protein B2 tracks with cytoplasmic tubulin.

While the peak locations of protein A and B2 were distinct, the edges of their distributions overlapped (Figure 2), which might reflect the resolution limits of the imaging, or be consistent with the observations of Aliyari et al. [32] that proteins B2 and A could be co-immunoprecipitated from lysates of FHV-infected *Drosophila* cells, or both. We therefore performed co-immunoprecipitation analyses with varied combinations of FHV-infected or uninfected cells, and immunoprecipitation and detection antibodies against protein A or B2, as shown in Figure 3A. The results confirm that proteins A and B2 co-immunoprecipitate from infected cells with antibodies against either, but that only subsets of each protein are pulled down. Since protein A and B2 both bind RNA [11,13] and might be pulled down together through independent interaction with common RNA strands, we used micrococcal nuclease—which digests single-stranded and double-stranded RNA and DNA—to deplete nucleic acids prior to co-immunoprecipitation. The lack of any significant difference in protein A and B2 co-precipitation in the presence or absence of RNA implies that protein A and B2 interaction is not mediated by nucleic acid or, at least, once established no longer requires continued RNA interaction (Figure 3A). 

To obtain a more precise and independent measure of the fractions of protein A and B2 that associate in infected cells, we further tracked these proteins in subcellular fractions obtained during isolation of mitochondria from FHV-infected *Drosophila* S2 cells using the same Qiagen Qproteome mitochondria isolation kit used in this study for cryo-EM sample preparation (see below). While nearly all protein A segregated with mitochondria, only ~8% of the total B2 in infected cells co-purified with mitochondria (Figure 3B). Taken together, these results corroborate both earlier reports and resolve their seeming conflict. Protein B2 exists in at least two pools: one small subset of B2 that interacts with and co-immunoprecipitates with protein A, and a second, much larger subset of B2 that does not interact with protein A.

### 3.2. Protein B2-Independent and RNA2-Independent Viral RNA Replication in Mammalian Cells

**Figure 4:**
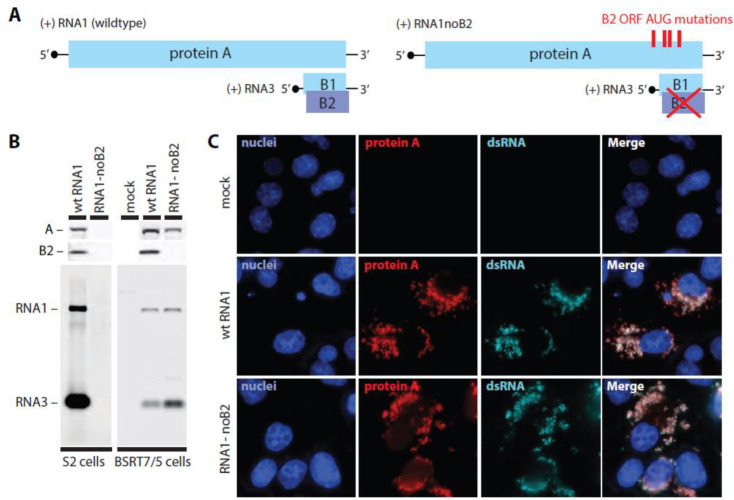
Analysis of the replication of wt FHV RNA1 and its mutant derivative RNA1-noB2 replication in insect and mammalian cells. (**A**) Schematic maps of wildtype RNA1 and an RNA1-derivative that lacks coding potential for B2 due to mutations of initiator and downstream methionine codons that are silent in the -1 frame encoding protein A sequence. (**B**) B2 is a strong suppressor of RNA-interference (RNAi), and RNA1-noB2 does not support stable accumulation of RNA1 and subgenomic RNA3 and protein A in *Drosophila* S2 cells that have a strong antiviral RNAi response (left lanes). By contrast, RNA1-noB2 launches RNA1 and RNA3 replication levels very similar to wildtype RNA1 in mammalian BSRT7/5 cells that do not display robust RNAi action (right lanes). (**C**) Fluorescence images of BSRT7/5 cells confirm accordingly that B2 is not needed and show that protein A and dsRNA replication intermediates are observed at the mitochondria of wildtype RNA1 and RNA1-noB2 transfected cells at similar levels and distribution.

As a basis to determine if any subdomains in the crown structure from fully infected cells might be contributed by B2 or capsid protein, we generated plasmids to launch FHV RNA1 from the baculovirus IE1 promoter in *Drosophila* S2 cells or from the bacteriophage T7 promoter by in vitro transcription or by DNA transfection into mammalian BSRT7/5 cells, BHK21-derivatives that express T7 RNA polymerase [34]. These plasmids were designed to launch either wildtype RNA1 or a mutant RNA1-derivative, designated RNA1-noB2, that no longer expresses protein B2 because the B2-initiating and multiple downstream in-frame B2 AUG codons were changed to threonine codons (Figure 4A). These changes are translationally silent in the overlapping amino acid coding sequence for protein A. Similar RNA1 derivatives have previously been shown to self-replicate in mammalian cell lines, which typically lack the strong antiviral RNAi response observed in insect cells [11] that is counteracted by B2 [5]. Omitting FHV RNA2 from these transfections ensured that capsid protein was absent. Transfecting either the T7 promoter plasmids directly or their in vitro RNA transcripts launched RNA1 replication in BSRT7/5 cells, but transfecting in 5’ capped vitro transcripts launched replication more efficiently and in many more cells and was generally used. The lower efficiency of direct DNA launching may be because the cytoplasmic RNA transcripts produced from transfected plasmids by T7 RNA polymerase in BSRT7/5 cells are not 5’ capped and likely are both inefficiently translated and rapidly degraded. 

Northern blots with RNA extracted from transfected cells were probed for viral RNA1 and RNA3 and showed that, as expected, RNA1-noB2 did not support detectable RNA1 replication in insect cells (Figure 4B). However, RNA1-noB2 launched RNA1 replication as well and often better than wildtype RNA1 in mammalian cells (Figure 4B). Western blotting confirmed RNA1 replication-dependent accumulation of protein A and the expected presence or absence of protein B2 in the cell lysates (Figure 4B). Immunofluorescence microscopy showed that, in transfected BSRT7/5 cells, mitochondrially associated protein A and dsRNA replication intermediates co-accumulated with similar distribution and equivalent levels regardless of the presence or absence of B2 (Figure 4C).

### 3.3. RNA Replication Complex Crowns Formed in the Absence of Protein B2 and Coat Protein Are Indistinguishable from Wildtype Nodavirus Crowns

**Figure 5:**
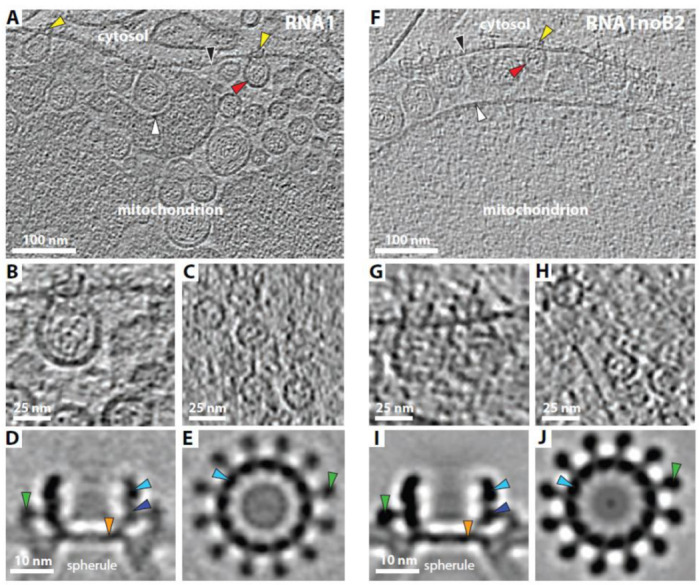
Cryo-ET of nodavirus RNA replication complexes in mammalian cells. (**A**,**F**) Indistinguishable spherules are induced on the outer mitochondrial membrane in mammalian BSRT7/5 cells transfected with either wildtype nodavirus RNA1 (**A**) or with RNA1-noB2 (**F**). Arrowheads: black = outer mitochondrial membrane; white = inner mitochondrial membrane; red = spherules, filled with dsRNA; yellow = crown-like structures at the spherule open necked connections to the cytoplasm. (**B**,**G**) Close-up side views of spherules with crowns visible in the plane of sectioning. (**C**,**H**) Close-up top views of crowns. (**D**,**I**) Cross sections of EM density maps of subtomogram-averaged side views of crowns. Arrowheads: light blue = apical lobe; dark blue = basal lobe; green = leg; orange = floor. (**E**,**J**) Cross sections of EM density maps of subtomogram-averaged top views of crowns with blue arrowheads pointing to the central crown turret and green arrowheads to the extended legs. Tomogram movies corresponding to panels A and F and Fourier Shell Correlation (FSC) curves corresponding to panels (**D**,**E**,**I**,**J**) are provided in Supplementary Movies S1 and S2 and Supplementary Figure S1.

To determine the effects of omitting protein B2 and capsid protein on the ultrastructure of nodavirus RNA replication crowns, mitochondria from mammalian BSRT7/5 cells transfected with RNA1 or RNA1-noB2 were purified, plunge-frozen and imaged using cryo-ET. The resulting tomograms showed that mitochondria from both wildtype RNA1-transfected cells (Figure 5A–C and Movie S1) and RNA1-noB2-transfected cells (Figure 5F–H and Movie S2) closely resembled all infection-specific features of mitochondria from FHV-infected Drosophila S2 cells (Figure 1B and references [23,24]). Parallels among all three cases included dilation of the space between the outer and inner mitochondrial membranes, filling of this dilated space with numerous RNA replication vesicles invaginated from the OMM with densely coiled fibrils inside, and crown-like protein densities at the necked apertures of these vesicles to the cytosol. 

For more detailed inspection, subtomogram averages of the crowns from wt RNA1- and RNA1-noB2-transfected BSRT7/5 cells were generated and compared. Cross-sections of the side (Figure 5D vs. Figure 5I) and top (Figure 5E,J) views of these crowns were not significantly distinguishable from each other or from crowns previously imaged from FHV-infected *Drosophila* cells [23,24]. Side views of these mammalian cell crowns with and without B2 displayed equivalent, ~19 nm diameter, ~14 nm high central turrets with basal and apical domains, legs extending radially outside of the central turret, a floor below the central turret, and, below the crown on each side, the membrane vesicle neck with a curvature precisely defined by binding to the two rings of membrane-interacting domains at the exterior radii of the crown floor and the outer legs (Figure 5D,I; compare also to [24]). Top views of these crowns confirmed their 12-fold symmetry and the dimensions and organization of the legs, central turret and crown floor (Figure 5E,J; again please compare to [24]).

**Figure 6:**
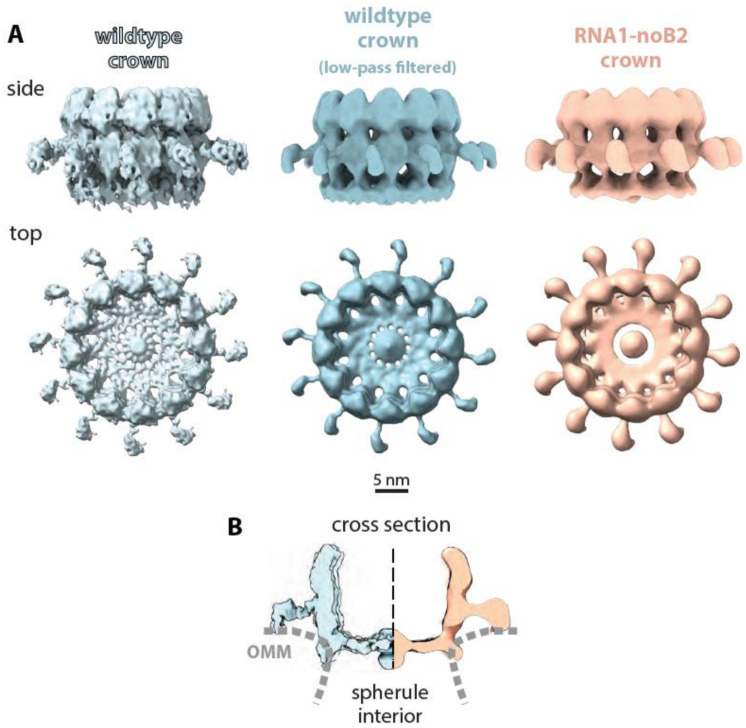
Comparison of nodavirus RNA replication complex crowns formed in nodavirus-infected insect cells to those formed in mammalian cells in the absence of Protein B2 and genomic RNA2. (**A**) On the right, light orange side and top view presentations of RNA1-noB2 replication-induced crowns in mammalian cells display all of the hallmark 12-fold symmetrical features of wildtype FHV infection-induced RC crowns shown in the published high-resolution crown image [24] on the left and the matched low-pass gaussian-filtered, slightly lower resolution version in the center. As shown, these common features include the apical and basal lobes of the central turret, inner floors with similar diameter central openings and central densities, and leg-like radial extensions. (**B**) Cross-sectioned comparison to further illustrate the matching shapes and membrane interactions of the high-resolution wildtype crown (leftmost images in panel (**A**)) and the RNA1-noB2 crown structures. OMM = outer mitochondrial membrane.

To further examine the structure of RNA1-noB2 crowns, we compared this 19 Å resolution RNA1-noB2 crown to the even higher resolution crown structure derived by particularly intensive imaging of FHV-infected S2 cells [24], which was used as the best subtomogram-averaged crown standard available. While the lower resolution of the RNA1-noB2 crown (Figure 6A,B, right side) does not reproduce the detailed surface variations of the wildtype crown from infected cells (Figure 6A,B, left side), the RNA1-noB2 crown displays all of the latter’s hallmark features in even greater detail than Figure 5I,J. These include again the apical and basal domain 12-member rings, 12 legs extending to ~35 nm diameter, and a lower floor that extends across the vesicle neck and includes a distinct, thicker central density.

As an additional comparison, we used low-pass filtering with the ChimeraX vop gaussian filter function to slightly reduce the displayed resolution of the wildtype crown to better match that of the RNA1-noB2 crown. The resulting slightly smoothed central image in Figure 6A further highlights the consistency of the two crown structures. In addition, Figure 6B compares a cross section of the RNA1-noB2 crown at right with a cross section of the high-resolution wildtype crown at the left. Most importantly, despite the absence of B2 and capsid proteins from the transfected BSRT7/5 cells, and whether compared to the high- or low-resolution wildtype images, the side, top and cross-sectional views of Figure 6 show that the RNA1-noB2 crown lacks none of the features of the wildtype crown from fully infected cells. This is highly meaningful since B2 forms a dimer ~5.5 nm long and 2.5 nm wide [35], which is similar in size to the crown legs and whose loss would be easily visible in this structure, and the capsid protein is significantly larger [36].

Thus, the results show that the nodavirus B2 and capsid proteins are not stable components of the isolated, 12-fold symmetric, protein A-containing crowns present on active FHV RNA replication complexes. Moreover, crowns from full FHV infection or partial FHV transfection and from insect and mammalian cells were all indistinguishable, further emphasizing the wide functional competence of FHV, and the potential to study and extend its applications in many cell types. The Discussion below considers the significance of these findings in relation to other results. 

## 4. Discussion

### 4.1. Nodavirus Protein A Is the Only Viral Protein Required for Stable, Active RNA Replication Crowns

Prior cryo-ET of mitochondria from nodavirus-infected cells revealed that in nodavirus genome RCs, the sole viral RNA replication factor, multifunctional protein A, resides in a stable, 12-fold symmetric, ringed crown surmounting the cytosolic side of the replication vesicle neck (Figure 1 and references [23,24]). The resulting crown structure is functionally relevant since the RCs on such isolated mitochondria remain active in RNA synthesis at levels similar to those in infected cells ([23,24], and unpublished results).

Determining the full protein composition and organization of these stable, synthetically active crowns is essential to defining their operation and the mechanistic pathways of viral RNA replication. As introduced above, the prime candidates for additional nodaviral proteins in crowns had been the B2 and capsid proteins due to their respective links to protein A and to RNA replication noted above. However, the results presented here show that simultaneously omitting B2 and capsid proteins did not result in loss or detectable alteration of any portion of the crown structure, even though these proteins are equivalent to or greater in size than major crown features such as the legs, and their removal would be readily detected (Figure 5 and Figure 6). Thus, not only is protein A the sole viral protein component of the wildtype RC crown, but the crown’s formation, structural conformation and RNA synthesis activity (Figure 4B) were not detectably influenced by the presence or absence of other viral proteins. 

### 4.2. Potential Transient Interactions with the Crown

The results presented here show that B2 and capsid proteins are not stable components of the crown and are not required for its assembly or RNA replication functions. Moreover, although as noted above B2 dimers are approximately as large as the highly prominent leg domains of crowns and FHV capsid protein is significantly larger [35,36], no remotely comparable deviations from the C12-symmetric structure of active, full crowns on mitochondria from FHV-infected cells were revealed when the cryo-ET imaging of these structures was analyzed without any imposed symmetry (Figure S2 of [24]) or by 2D- and 3D-classification of crown image populations into subclasses ([24] and unpublished results). Thus, neither B2 nor capsid proteins have been detected even as sub-stoichiometric components of the active, 12-fold symmetric protein A crowns produced in normal infections.

While B2 and capsid proteins are not stable crown components or required for crown functions in RNA replication, these proteins might interact with the crown transiently. Such transient interaction seems particularly possible at later stages of crown function, to facilitate the transfer of newly synthesized RNA products from the crown to downstream processes such as translation and encapsidation. Support for such orchestrated hand-offs comes in part from coronavirus studies. Coronavirus RNA replication occurs in double membrane vesicles bearing 6-fold symmetric “crown” channels, which contain viral protein nsp3 and, similarly to nodaviral crowns, appear to coordinate synthesis and release of new viral positive-strand RNAs [37]. The cytosolic side of coronavirus crowns interact dynamically with large assemblies that appear likely to include viral nucleoprotein N, which binds crown component nsp3 and may use this association to facilitate capture and encapsidation of new positive-strand RNAs [37]. Similarly, multiple results indicate functional links between nodavirus RNA replication and encapsidation [8,31,38] and transient nodavirus capsid protein interaction with its crown could promote encapsidation of nascent RNA products. 

In parallel, transient interaction of B2 with the crown could facilitate B2’s known role of binding dsRNA segments to protect them from innate immune recognition [35]. Such a dynamic is potentially consistent with our finding (Figure 2 and Figure 3) that B2 exists in at least two distinct pools: a minor pool (~8%) associated, presumably transiently, with protein A, and a larger pool not bound to protein A but accumulating in its vicinity, possibly bound to dsRNA segments of viral RNA products accumulating near RCs in the surrounding, dense viroplasms that support virion assembly [30]. Possible additional B2 functions relating to its RNA binding might include assisting in recruiting genomic RNA2 replication templates that, unlike RNA1, can only be supplied to the RC in trans and initially localize to other sites [39], or assisting in assembling genomic RNA1 and RNA2 (Figure 1A) for their co-encapsidation, which appears tightly linked to RNA replication [38].

Present results do not rule out host proteins as possible crown components or interactors, but such host factor interactions might also be transient in many cases. For example, RC formation by many positive-strand RNA viruses involves recruiting host factors involved in membrane synthesis or rearrangement but, once RC assembly is complete, most such activities do not appear—and in some cases are known to not be - retained at RCs or required for RC function [40,41,42]. Additionally, one or more host translation factors might bind the crown transiently to be poised for capturing nascent viral positive-strand RNAs for translation, just as capsid protein might temporarily bind the crown to guide new viral RNAs into encapsidation. 

### 4.3. Concluding Remarks

Nodavirus protein A (998 aa) is an amazingly multifunctional protein that targets mitochondrial membranes [14,18], selectively recruits viral genomic RNA templates [16,17], provides the polymerase and RNA 5’ capping functions for RNA synthesis, and possesses multiple independent multimerization domains [9]. These extensive functions are now further expanded by the finding that protein A is the sole viral protein required for assembly of wildtype crowns and RCs, and the sole stable viral protein component in crowns. It is also notable that FHV crowns induced by normal infection of insect cells or transfection of mammalian cells were indistinguishable (Figure 6). This is both consistent with the central role of viral protein A in inducing crowns and, together with the FHV genome’s ability to direct genome replication and virion production in nearly any eukaryotic cell [4,5,6], shows that any host factors required must be highly conserved. Although nodavirus studies only recently revealed the existence of crowns on positive-strand RNA virus RCs, emerging results with alphavirus and coronavirus RCs have already disclosed similar proteinaceous portals whose growing parallels imply that many fundamental features of crown structure and function are widely preserved across positive-strand RNA viruses [8,37,43,44,45]. Rapidly advancing understanding of the structure, assembly and operation of such crowns promises to continue illuminating their central roles in organizing not only RNA replication but also key events in additional stages of infection and virus–host interactions.

## Data Availability

The cryo-EM tomography maps of the RNA1-noB2-derived crowns and wildtype RNA1-derived crowns from BSRT7/5 cells are available in the wwPDB EM Data Bank (EMDB) under accession codes EMD-28995 and EMD-28996, respectively.

## Supplementary Materials

The following supporting information can be downloaded at: https://www.mdpi.com/article/10.3390/v14122711/s1, Figure S1: FSC plots for Figure 5; Movies S1 and S2: Tomography movies for Figure 5.
