# Supplementary material for: Multifunctional Protein A Is the Only Viral Protein Required for Nodavirus RNA Replication Crown Formation
Source: Viruses. 2022 Dec 3;14(12):2711. doi: 10.3390/v14122711 (PMC9788154; doi:10.3390/v14122711)
Supplement: Supplementary file 1 [file viruses-14-02711-s001.zip › Supplementary Figure S1_den Boon et al_revised.pdf]

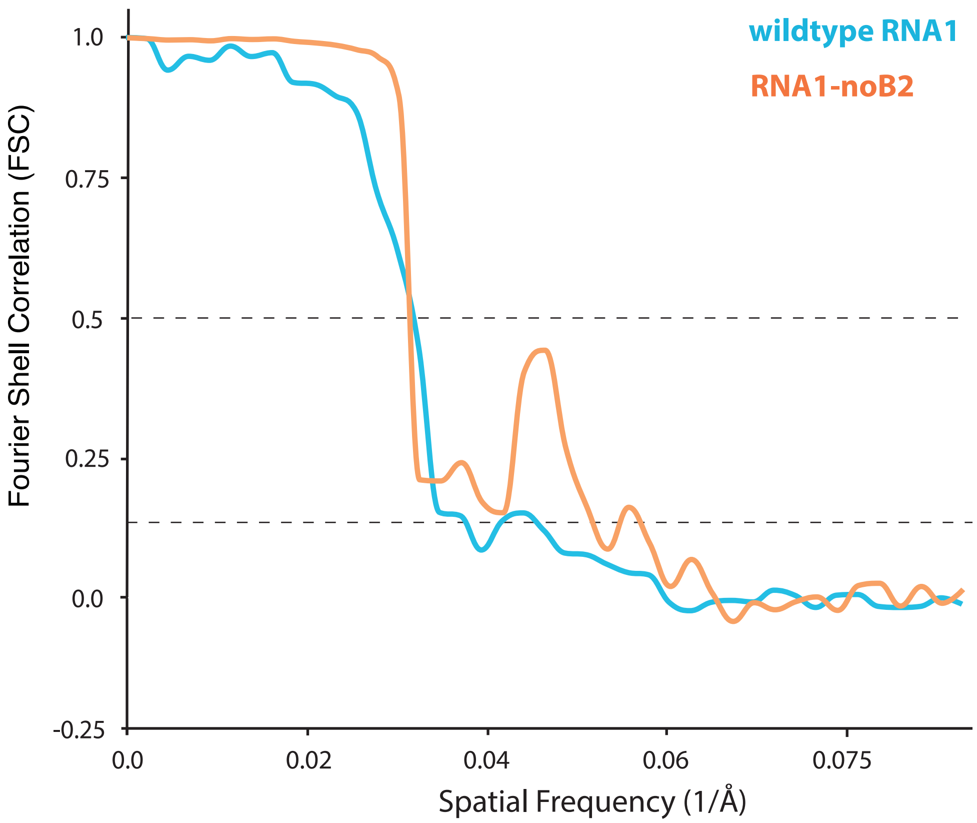

Supplementary Figure S1. Fourier Shell Correlation curves with FSC 0.5 and 0.143 thresholds (dashed lines) indicating the resolution of the 12-fold symmetry-imposed structures for wildtype RNA1-induced crowns (blue) and RNA1-noB2-induced crowns (pink) corresponding to the EM density maps displayed in Figure 5 panels D, E, I, and J.
